# An investigation of the direct and indirect effects of desire for independence and perceived level of achievement on singlehood status

**DOI:** 10.1038/s41598-024-75086-w

**Published:** 2024-10-11

**Authors:** Menelaos Apostolou, Antonios Kagialis, Timo Juhani Lajunen

**Affiliations:** 1 Department of Social Sciences, University of Nicosia, 46 Makedonitissas Ave, Nicosia, 1700 Cyprus; 2 Department of Psychiatry, School of Medicine, University of Crete, Heraklion, Crete Greece; 3 Department of Psychology, Norwegean University of Science and Technology, Trondheim, Norway

**Keywords:** Singlehood, Voluntary singlehood, Involuntary singlehood, Desire for independence, Perceived level of achievement, Psychology, Human behaviour

## Abstract

The present study aimed to examine the associations between the desire for independence, perceived level of achievement, and relationship status. Specifically, we conducted the study with a sample of 667 Greek-speaking participants (389 women, 275 men, and three participants who did not indicate their sex) who were either single or in an intimate relationship. We employed close-ended questionnaires that included instruments developed using AI. We found that men and women who desired more independence were more likely to be voluntarily single than in an intimate relationship. Additionally, a higher desire for independence was associated with more years being single, but this effect was significant only for men. Furthermore, we found that the perceived level of achievement was not significantly associated with relationship status directly; however, higher scores in this dimension were associated with fewer years spent as single for men. Moreover, a higher perceived level of achievement was associated with a decreased probability of being involuntarily single rather than in an intimate relationship and fewer years spent as single indirectly, by being associated with higher self-esteem, which was associated with higher flirting capacity. These paths were significant for both men and women. Our findings suggest that the desire for independence and perceived achievement play an important role in explaining why some people are single and others in an intimate relationship.

## Introduction

Singlehood, that is, not having an intimate partner, constitutes an increasingly common state, especially in Western societies^1–3^. For instance, one study found that 47% of adults under 30 in the USA were single (https://www.pewresearch.org/short-reads/2023/02/08/for-valentines-day-5-facts-about-single-americans/?utm_content=buffer0ee01&utm_medium=social&utm_source=twitter.com&utm_campaign=buffer). The increasing commonness of the phenomenon is puzzling. For instance, in an evolutionary perspective, mating and reproduction, are key in evolutionary success^4^, so we would expect that most people would not be single. Moreover, singlehood is associates with negative emotions such as loneliness^5^, and so many singles consider their status undesirable^6^. Understanding the high prevalence of the phenomenon and finding ways to address it when is considered undesirable, requires identifying its causes. Recent research effort have identified several factors which are associated with singlehood^7^. The purpose of the current study is to extend the existing literature by examining the association of desire for independence and perceived level of achievement with singlehood status. We will start developing our argument by examining the different types of singlehood and their occurrence.

## Types of singles

Not all singles are single for the same reason^5,8^. Three main categories of singles have been recognized: People who are single because they face difficulties in attracting mates (involuntarily singles), those who prefer to be single (voluntarily singles), and those who have recently exited a relationship and have not had sufficient time to find another mate (between relationships singles)^6^. A cross-cultural study employed a sample of 7,181 participants from 14 different post-industrial nations and found that about 13% of the participants indicated that they were involuntarily single, more than 15% indicated that they were voluntarily single, and 10% were between-relationships single^9^. It has also found that all types of singlehood were more prevalent in younger age groups, with variation in prevalence across different cultures.

These findings raise the question of what factors predict the different types of singlehood. For instance, why do some people face difficulties attracting mates and are thus more likely to be involuntarily single, while others prefer not to be in a relationship and are thus voluntarily single? The existing literature has identified several predictors of singlehood. More specifically, people who have poor flirting skills, face sexual difficulties, are very choosy, and experience low self-esteem are more likely to be involuntarily single than in an intimate relationship^7^. On the other hand, people who had past negative experiences with intimate relationships and who scored high in Dark Triad personality traits, are more likely to be voluntarily single than in an intimate relationship^10,11^. Qualitative and quantitative studies asking singles about the reasons why they were in this status have found desire to be independent, and not having achieved much in life to be relatively common responses^12,13^. Accordingly, the current study aimed to add to the existing literature by examining whether desire for independence and perceived level of achievement are associated with singlehood status.

### Desire for independence

When in an intimate relationship, people need to consider the impact that their actions would have on their intimate partners and avoid engaging in acts that would trigger adverse reactions. For instance, to ensure a peaceful relationship, people need to refrain from flirting or having sex with individuals other than their legitimate partners. Moreover, people need to some degree to conform to and attempt to satisfy their partners’ wishes even if they do not comply with their own. For example, people may accompany their partners on a night out although they would prefer to stay home resting. It follows that forming intimate relationships could lead to a lack of freedom and independence to do as one wishes. Consistent with this argument, when people were asked to indicate what they considered a disadvantage of being in an intimate relationship, they indicated lack of independence to be a prominent disadvantage^14^.

The disadvantages of being in an intimate relationship are advantages of being single. Single people have more freedom and independence because they do not need to adjust their actions to the wishes, needs, and sensitivities of their intimate partners. In accordance with this argument, when asked to indicate the benefits of being single, individuals nominated independence to be a primary one^15^. People vary in most psychological dimensions^16^, and so it is reasonable to argue that they also vary in their desire for independence. That is to say, some people have a higher desire to lead an independent life while others do not mind giving up some of their independence. Those who score high in desire for independence would find it more difficult to stay in an intimate relationship and may consider singlehood to be a better option. Accordingly, we predict that a higher desire for independence would be associated with an increased probability of being voluntarily single rather than in an intimate relationship (H_1_).

Consistent with this prediction, when people were asked to indicate why they were single, they indicated independence as a reason. More specifically, one study analyzed 6,794 responses from a Reddit thread asking men why they were single and classified them into 43 categories^12^. The fourth most frequent category was “Not interested in relationships,” which involved reasons such as “I like my freedom and privacy.” Another study asked 6,822 singles from eight different countries to rate 92 reasons that have driven them to be single^13^. Using exploratory factor analysis, the researchers classified these reasons into 12 categories, one being “I want to be free to do whatever I want.” This factor included reasons such as “I want to not have to answer to anyone about what I am doing” and “I do not want to lose my freedom,” and was rated among the most important ones for being single.

## Level of achievement

Success and achievements in different domains of life, such as getting into a good university, signal abilities including one’s capacity to generate resources^17^. Achievements in different domains of life, such as securing a good job, also make resources available that individuals can direct in supporting their intimate partners and their children. In effect, success and achievements are considered highly desirable in a mate^4^. For instance, research on mate preferences finds that people prefer mates who have a good job, enjoy a good social status, and have good education^18–20^.

It follows that having achieved much in life would make it easier for people to attract mates and thus more likely to secure one. This leads to the hypothesis that achievements would have a direct effect on relationship status, with a lower level of achievement being associated with an increased probability of being involuntarily single (H_2_). Consistent with this prediction, when people were asked why they were single, they indicated their poor level of achievement. In particular, in the Reddit study discussed above, one group of reasons for being single was the “Lack of achievements,” which included items such as “Because I’m a 41-year-old with all the qualifications and achievements of a 19-year-old”^12^. This was a commonly reported reason for being single, found in the middle of the hierarchy of occurrence (22/43). Similarly, in the cross-cultural study on the reasons for being single, the “Poor achievement record” emerged, which involved reasons such as “I have not achieved much in life and I do not think I am attractive as a mate” and “My financial situation prevents me from having a relationship”^13^. About one in four participants indicated this to be an important reason why they were single.

The level of achievement may also have an indirect effect on relationship status through self-esteem and flirting capacity. More specifically, it has been argued that self-esteem, or one’s perceived worthiness as a person^21^, constitutes a mechanism that acts as a sociometer, informing people about their standing in the world^22–24^. In this respect, life achievements would boost, and lack of such achievements would impair one’s self-esteem. Self-esteem has been found to predict relationship status predominantly through flirting capacity. In particular, low self-esteem has been associated with lower flirting capacity, which in turn, has been associated with an increased probability of being involuntarily single^25^. Accordingly, we predict that there would be an indirect effect of achievement on relationship status through self-esteem and flirting capacity: Lower levels of achievement would be associated with lower self-esteem, which would be associated with lower flirting capacity, which would be associated with an increased probability of being involuntarily single (H_3_).

## Sex differences

Above, we have argued that the desire for independence and the perceived level of achievement constitute predictors of voluntary and involuntary singlehood, respectively. However, the effect of these two factors may differ for men and women. Specifically, men’s reproductive success is positively related to the number of different sexual partners they can access, which is not the case for women. Accordingly, men tend to prefer sexual relationships without commitment more than women do^4^. Given this, men may desire to be single more than women to have the freedom to engage in casual sex. Furthermore, research on mate preferences finds that women place higher importance than men on the social status and career success of a prospective mate^4^. Thus, it could be the case that a low level of achievement could be more impairing to men’s than women’s capacity to attract mates.

The arguments above indicate that the desire for independence and perceived level of achievement may affect the singlehood status of men and women differently. Existing literature suggests that this is the case for other predictors of singlehood. For instance, one study examined the association of 17 different variables with involuntary singlehood and found that, in most cases, the associations were different for men and women^7^. Accordingly, to examine whether this is the case for our variables of interest, in the current research, we examined the predicted associations on the pooled sample as well as individually for men and women.

## The current study

Studies that asked people why they were single have found some support for the hypotheses that the desire for independence and the perceived level of achievement are associated with singlehood. However, these findings are not sufficient for testing these hypotheses. To see why, we may consider the following example: When asked why they were single, people indicated that one reason was their lack of achievements in life. Yet, this finding could be due to people who have achieved less being less attractive as mates, or their low level of achievement impairing their flirting capacity by lowering their self-esteem, or both. Accordingly, additional research is necessary to measure how people score in the dimensions of interest and examine if their scores are associated with relationship status, which is the purpose of the current study. In particular, our study aimed to test three hypotheses:

### H1

Higher desire for independence would be associated with an increased probability of being voluntarily single rather than in an intimate relationship.

### H2

Perceived level of achievement would have a direct effect on relationship status, with a lower level of achievement being associated with an increased probability of being involuntarily single rather than in an intimate relationship.

### H3

**Fig. 1:**
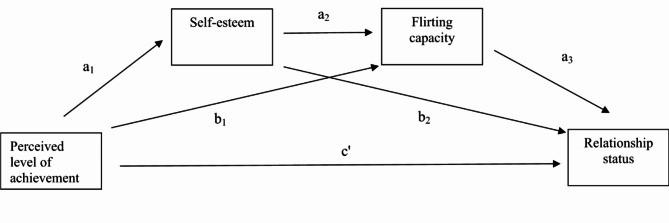
The figure above depicts the direct and indirect pathways that perceive achievement is associated with relationship status through self-esteem, and flirting capacity.

Lower perceived level of achievement would be associated with lower self-esteem, which would be associated with lower flirting capacity, which would be associated with an increased probability of being involuntarily single rather than in an intimate relationship (the predicted association is depicted in Fig. 1).

These predictions would also hold for years being single. In particular, we predict that a higher desire for independence would be associated with more years being single, and a lower level of perceived achievement would be associated with more years being single both directly and indirectly through being associated with lower self-esteem and lower flirting capacity.

## Methods

### Participants

The study was conducted at a private university in the Republic of Cyprus and received approval from the university’s ethics review board. Participants were recruited by forwarding the study link to students and colleagues with a request to share it within their social network, and by promoting it on social media platforms (Facebook, Instagram) targeting participants residing in Greece and the Republic of Cyprus. In total, 667 Greek-speaking individuals (389 women, 275 men, and three participants who did not indicate their sex) participated in the study. The average age of the women was 36.0 years (*SD* = 13.4), and the average age of the men was 41.3 years (*SD* = 13.3). Furthermore, 25.3% of the participants indicated that they were in an intimate relationship, 25.3% were married, 18.4% were involuntarily single, 16% were between-relationships single, 8.5% were voluntarily single, and 6.3% indicated their relationship status as ‘other.’ In addition, single participants reported being single for an average of 4.3 years (*SD* = 6.8). The present study was approved by the University of Nicosia, Department of Social Sciences Ethics Review Board, and all methods were carried out in accordance with ethical guidelines and regulations. Informed consent was obtained from all participants. All data are available here: https://osf.io/jkzyb/?view_only=f8d0d6746bfc4f36a5903886f8e627f3.

## Materials

The study was conducted in Greek, run online, designed using Google Forms, and consisted of five parts. In the first part, we measured desire for independence; in the second part, we measured perceived level of achievement; in the third part, we measured flirting capacity; and in the fourth part, we measured self-esteem. In the fifth part, we recorded demographic information, including biological sex, age, and relationship status. Relationship status included the following categories: “In a relationship,” “Married,” “Involuntarily single: I want to be in a relationship, but I find it difficult to attract a mate,” “Single between-relationships: My relationship has recently ended and I have not yet found another partner,” “Prefer to be single: I am not interested in being in a relationship,” and “Other.” Participants who reported that they were single, were subsequently asked to report for how many years they had not been in an intimate relationship. The order of presentation for each question in each part was randomized across participants.

To develop a measure of desire for independence, we followed this procedure^26^: We utilized AI tools (Chat GPT version 3.5 and Google Gemini) to generate items that could be used in a psychometric instrument to measure such desire. From the generated items, we selected 11 with the highest face validity. Based on this choice, we constructed a first version of the instrument and piloted it on a sample of 25 students. We conducted an internal consistency analysis using Cronbach’s alpha and dropped two items that reduced overall consistency. The final instrument consisted of nine items such as “I wouldn’t trade my independence for anything”, and “I highly value my personal freedom and independence”, and is presented in Appendix. A higher mean score indicated more perceived choices. The Cronbach’s alpha for this instrument was 0.86. Moreover, we applied Item Response Theory (IRT) in order to assess the quality of the final scale. In particular, we fitted a polytomous Rasch model using Marginal Maximum Likelihood Estimation (MMLE). For this purpose, we employed the Jamovi software package version 2.6.2. Here, Person reliability was 0.79 indicating a moderate to high level of consistency. That is to say, our scale measured relatively reliably desire for independence across different participants. Using the same procedure, we developed a three-item instrument (e.g., “I have achieved many things in my life”) to measure perceived level of achievement (Appendix B). In this case, the Cronbach’s alpha was 0.79. We applied Item Response Theory (IRT) as above, with Person reliability being 0.70.

We also performed a multigroup confirmatory factor analysis to examine metric invariance across sexes for the two instruments we have developed. Metric invariance means that each item contributes to the latent construct to a similar degree across groups. Initially, an unrestricted model was fitted, and subsequently, the same model was fitted with the factor loadings constrained to be equal across sexes. If the fit of the model did not substantially worsen compared to the unrestricted model, we could conclude that the factor loadings were equivalent across sexes. With respect to desire for independence, the Chi-square difference test (Δχ² = 13.06, Δdf = 8) was not significant (*p* = .110), which was also the case for the perceived level of achievement (Δχ² = 2.84, Δdf = 2) (*p* = .242). These result indicate that our instruments measure the constructs in the same way for both men and women.

Furthermore, we measured flirting capacity using a seven-item instrument developed in previous research^27^. Scores could range from “1” to “5,” with a higher score indicating higher flirting capacity. Cronbach’s alpha for this scale was 0.89. Additionally, we measured self-esteem using the Rosenberg Self-Esteem Scale, which consisted of 10 items with a higher score indicating higher self-esteem^21^. The Cronbach’s alpha for this scale was 0.89.

### Data analysis

In order to test H_1_ and H_2_, we performed multinomial logistic regression, where relationship status was the dependent variable, and desire for independence, perceived level of achievement, self-esteem, flirting capacity, participants’ sex, and age were the independent variables. Regarding the sex variable, the responses of participants who did not indicate their sex were not included as a separate category as they were too few. Consequently, these responses were excluded from the analysis. Moreover, we performed an ANCOVA test, where years single was the dependent variable, participants’ sex was the categorical independent variable, and desire for independence, perceived level of achievement, self-esteem, flirting capacity, and age were the independent continuous variables. Both analyses were also performed separately for men and women by dropping the sex independent variable.

In order to test H_3_, that is to identify the direct and indirect effects of achievement on relationship status (Fig. 1), we conducted a serial mediation analysis. Specifically, relationship status was the dependent variable, perceived level of achievement was the independent variable, self-esteem and flirting capacity were the mediators, and sex and age were covariates. In this analysis, perceived level of achievement would be associated with self-esteem, which in turn would be associated with flirting capacity, which would be associated with singlehood status. For running the mediation, we created a new relationship status variable with two levels, namely “in an intimate relationship” (*N* = 338) that included the “in a relationship” (*N* = 169) and the “married” (*N* = 169) categories, and the involuntarily single (*N* = 123) category. We performed the analysis for the pooled sample as well as separately for men and women. In all cases, unstandardized indirect effects were calculated for each of the 10,000 bootstrapped samples, and the 95% confidence interval was computed by determining the indirect effects at the 2.5th and 97.5th percentiles. The analysis was performed using SPSS version 28 and the PROCESS version 4.2 macro.

## Results

### Preliminary analysis

In order to examine if there were sex and age differences in the desire for independence, we ran an ANCOVA test, where the desire for independence was the dependent variable, sex was the independent categorical variable, and age was the continuous independent variable. There was no significant main effect of age (*p* = .329), but there was a significant main effect of sex [*F*(1,630) = 5.50, *p* = .011, η_p_² = 0.010]. In particular, women gave higher scores (*M* = 3.17, *SD* = 0.61) than men (*M* = 4.04, *SD* = 0.68). We repeated the same analysis with the dependent variable being perceived level of achievement. The results indicated that there was no significant main effect of age (*p* = .070), but there was a significant main effect of sex [*F*(1,636) = 5.50, *p* = .009, η_p_² = 0.011]. More specifically, women gave higher scores (*M* = 3.75, *SD* = 0.88) than men (*M* = 3.58, *SD* = 0.93). Additionally, we correlated self-esteem and perceived level of achievement using Pearson’s product-moment correlation. We found a significant positive correlation between the two variables [r(665) = 0.64, *p* < .001 (two-tailed)]. A zero-order correlation matrix of all the independent variables is reported in the Appendix.

### Desire for independence

From Table 1, we can see that, for the pooled sample, there was a significant main effect of desire for independence on relationship status. More specifically, higher desire for independence was associated with a decrease in the likelihood of being in any group of relationship status versus being voluntarily single. For example, one unit increase in the desire for independence was associated with an 80% [(1–0.20) * 100] decrease in the probability of being married rather than voluntarily single. Note that each main effect controlled for all of the other variables in our model, so this 80% was specifically the case when one controlled for sex, age, perceived level of achievement, self-esteem, and flirting capacity. The effect was significant for both men and women, with the odds ratios indicating it to be more pronounced in men. For instance, one unit increase in the desire for independence was associated with a 59% [(1–0.41) * 100] decrease in the probability of being in a relationship rather than involuntarily single, with the respective decrease being 89% [(1-0.19) * 100] for men.

**Table 1:** Main effects and odds ratios on relationship status.

	Desire for independence		Perceived level of achievement		Self-esteem		Flirting capacity	
	*p*-value	OR	*p*-value	OR	*p*-value	OR	*p*-value	OR
**Pooled**	< 0.001		0.699		0.501		< 0.001	
Involuntarily single		0.29*		1.11		0.51		0.62*
Between relationships		0.22**		1.42		0.44		1.45
In a relationship		0.32*		1.23		0.66		1.33
Married		0.20**		1.27		0.70		1.20
Other		0.18**		1.01		0.58		0.95
**Women**	0.015		0.875		0.802		< 0.001	
Involuntarily single		0.50		1.24		0.46		0.77
Between relationships		0.29*		1.08		0.73		1.73
In a relationship		0.41*		1.06		0.65		1.55
Married		0.28*		0.87		0.82		1.86*
Other		0.23*		0.75		0.67		0.53
**Men**	0.003		0.233		0.481		0.001	
Involuntarily single		0.13*		1.23		0.52		0.37*
Between relationships		0.12*		2.13		0.31		0.86
In a relationship		0.19*		1.78		0.73		0.89
Married		0.11*		2.02		0.65		0.41*
Other		0.10*		1.62		052		0.77

Note. The reference category is voluntarily single.

*Significant at 0.05.

**Significant at 0.001.

From Table 2, we can see that, for the pooled sample, there was a significant main effect of desire for independence on the years being single. More specifically, one unit increase in desire for independence was associated with 1.97 more years spent as single. We can see further that the effect was only significant for men, with one unit increase in independence being associated with 2.55 more years spent as single.

**Table 2:** Main effects and regression coefficients on years single.

	Desire for independence			Perceived level of achievement			Self-esteem			Flirting capacity		
	b	*p*-value	η_*p*_^2^	b	*p*-value	η_*p*_^2^	b	*p*-value	η_*p*_^2^	b	*p*-value	η_*p*_^2^
Pooled	1.97	0.005	0.031	-1.71	0.004	0.034	1.49	0.164	0.008	-2.39	< 0.001	0.088
Women	0.66	0.407	0.006	-0.16	0.788	0.001	-0.60	0.571	0.003	-0.96	0.050	0.034
Men	2.55	0.016	0.045	-2.59	0.006	0.058	2.56	0.143	0.017	-3.34	< 0.001	0.114

### Perceived achievement

From Table 1, we can see that for the pooled sample, there was no significant main effect of perceived level of achievement on relationship status. This was also the case for men and women. We can see further that there was no significant main effect of self-esteem, but there was a significant main effect of flirting capacity. From Table 2, we can see that for the pooled sample, there was a significant main effect of perceived level of achievement on years single. However, the effect was significant only for men, where one unit increase in perceived achievement was associated with 2.59 fewer years spent as single. We can see further that there was no significant main effect of self-esteem, but there was a significant main effect of flirting capacity.

### Direct and indirect associations

From Table 3, we can see that there was a significant association between perceived level of achievement and self-esteem, with a higher level of achievement being associated with higher self-esteem. Moreover, there was a significant association between self-esteem and flirting capacity, with higher self-esteem being associated with higher flirting capacity. Additionally, flirting capacity was associated with relationship status. For instance, for the pooled sample, one unit increase in flirting capacity was associated with an 86% [(1.86–1) * 100] increase in the probability of being in an intimate relationship rather than involuntarily single. We can also observe that there was no significant direct association between achievement and relationship status. These findings held true for the pooled sample as well as for women and men.

**Table 3:** The direct effects estimated in serial mediation analysis results on relationship status.

	Achievement on relationship status (c’)	Achievement on flirting capacity (b_1_)	Achievement on self-esteem (a_1_)	Self-esteem on flirting capacity (a_2_)	Self-esteem on relationship status (b_2_)	Flirting capacity on relationship status (a_3_)
Pooled	1.07 (0.76–1.49)	0.07 (-0.04 − 0.19)	0.39** (0.34–0.43)	0.78** (0.58–0.97)	1.52 (0.83–2.76)	1.86** (1.41–2.45)
Women	0.83 (0.52–1.33)	0.03 (-0.13–0.18)	0.36** (0.30–0.41)	0.84** (0.59–1.10)	1.69 (0.73–3.90)	2.00** (1.39–2.87)
Men	1.57 (0.93–2.65)	0.17 (-0.02–0.35)	0.43** (0.36–0.50)	0.64** (0.34–0.94)	1.28 (0.53–3.08))	1.56* (1.00–2.43)

*Significant at 0.05, ** significant at 0.001.

Note. The (c’), (b_2_) and (a_3_) are in odds ratios. The reference category is involuntarily single.

From Table 4, we can see that the ‘achievement – self-esteem – flirting capacity’ pathway was significant for both the pooled and the individual samples. For instance, for the pooled sample, one unit increase in perceived achievement was associated with a 20% [(1.20–1) * 100] increase in the probability of being in an intimate relationship rather than involuntarily single, by being associated with higher self-esteem, which in turn was associated with higher flirting capacity. From Table 5, we can see that there was a direct effect of perceived level of achievement on years being single, but only for men. Moreover, from Table 6, we can see that the ‘achievement – self-esteem – flirting capacity – years single’ path was significant in all instances. For example, for the pooled sample, one unit increase in perceived achievement was associated with a 0.79-year decrease in the length of singlehood by being associated with higher self-esteem, which was in turn associated with higher flirting capacity.

**Table 4:** The indirect effects estimated in serial mediation analysis results on relationship status.

	Achievement * self-esteem (a_1_*b_2_)	Achievement * flirting capacity (b_1_*a_3_)	Achievement * self-esteem * flirting capacity (a_1_*a_2_*a_3_)
Pooled	1.17 (0.91–1.49)	1.05 (0.97–1.15)	1.20* (1.10–1.35)
Women	1.21 (0.90–1.70)	1.02 (0.90–1.16)	1.23* (1.10–1.46)
Men	1.11 (0.74–1.65)	1.08 (0.98–1.28)	1.13* (1.00–1.35)

*Significant at 0.05, ** significant at 0.001.

Note. All results depict odds ratios. The reference category is involuntarily single.

**Table 5:** The direct effects estimated in serial mediation analysis results on years single.

	Achievement on years single (c’)	Achievement on flirting capacity (b_1_)	Achievement on self-esteem (a_1_)	Self-esteem on flirting capacity (a_2_)	Self-esteem on years single (b_2_)	Flirting capacity on years single (a_3_)
Pooled	-1.54* (-2.70 to -0.37)	-0.07 (-0.22 to 0.08)	0.40** (0.34 to 0.46)	0.87** (0.62 to 1.11)	1.65 (-0.43 to 3.73)	-2.27** (-3.24 to -1.30)
Women	-0.09 (-1.27 to 1.08)	-0.17 (-0.41 to 0.06)	0.45** (0.36 to 0.53)	0.96** (0.60 to 1.32)	-0.48 (-2.50 to 1.54)	-0.94* (-1.086 to -0.03)
Men	-2.40* (-4.27 to 0.53)	0.01 (-0.19 to 0.20)	0.36** (0.29 to 0.44)	0.81** (0.47 to 1.14)	2.91 (-0.58 to 6.40)	-3.24** (-4.91 to -1.57)

*Significant at 0.05, **significant at 0.001.

**Table 6:** The indirect effects estimated in serial mediation analysis results on years single.

	Achievement * self-esteem (a_1_*b_2_)	Achievement * flirting capacity (b_1_*a_3_)	Achievement * self-esteem * flirting capacity (a_1_*a_2_*a_3_)
Pooled	0.66 (-0.05 to 1.43)	0.15 (-0.20 to 0.55)	-0.79* (-1.29 to -0.37)
Women	− 0.21 (-0.88 to 0.41)	0.16 (-0.06 to 0.50)	-0.41* (-0.82 to -0.10)
Men	1.06 (-0.10 to 2.33)	-0.02 (-0.71 to 0.68)	-0.95* (-1.86 to -0.27)

*Significant at 0.05, **significant at 0.001.

## Discussion

In the present study, we found that men and women who desired more independence were more likely to be voluntarily single than in an intimate relationship. In addition, a higher desire for independence was associated with more years being single, but the effect was significant only for men. We also found that the perceived level of achievement was not significantly associated with singlehood status; however, higher scores in this dimension were associated with fewer years spent as single for men. Moreover, in both sexes a higher perceived level of achievement was associated with a decreased probability of being involuntarily single rather than in an intimate relationship and fewer years spent as single, by being associated with higher self-esteem, which was associated with higher flirting capacity.

Consistent with our original prediction, participants did differ in their desire for independence, and this variation was associated with variation in relationship status and the years being single. In particular, participants who indicated a higher desire for independence were more likely to be voluntarily single than in other categories of relationship status. Previous research asking singles why they were not in an intimate relationship has found the desire for freedom to be a common reason^12,13^. In the current study, we found an association between people’s desire for independence and relationship status, providing additional evidence that the desire for freedom constitutes an important predictor of voluntary singlehood.

Moreover, we found that the observed association was more pronounced for men. One possible explanation is that men are more likely than women to follow a short-term mating strategy, that is, to look for casual mates^18^. Thus, although men and women did not differ on average in their level of desire for independence, the reasons why some men desire high independence may be different from the reasons why women desire high independence. In particular, men may desire more independence in order to be able to pursue casual mating, something that is less so for women, resulting in the desire for independence having a larger impact on the relationship status of men than of women. This could also explain why the desire for independence was significantly associated with years being single only for men. More research is required to investigate the reasons behind the observed associations.

In our argument, people who have a strong desire for independence are more likely to prefer being single to avoid the constraints of an intimate relationship. Accordingly, we developed an instrument to measure the general desire for independence. Yet, there may be domain-specific independence related to romantic relationships. Specifically, being in an intimate relationship may constrain individuals more in certain areas, such as the freedom to flirt or engage in sexual activities with others, and less in areas like career choices. Consequently, there may be individual differences where people score high in their desire for independence specific to romantic relationships but not necessarily in general independence. Future studies should aim to measure the desire for romantic relationship-specific independence and examine its association with singlehood status, as well as whether it is distinct from the desire for general independence.

Our prediction that there would be a significant direct association between perceived level of achievement and relationship status was not confirmed. Nevertheless, a higher perceived level of achievement was associated with fewer years being single for male participants. On the other hand, as we originally predicted, the perceived level of achievement was indirectly associated with relationship status and years being single: Higher perceived level of achievement was associated with higher self-esteem, which was in turn associated with higher flirting capacity, which in turn was associated with a decreased probability of being involuntarily single and fewer years spent as single. In previous studies, when people were asked why they were single, they reported a low level of achievement to be such a factor^12,13^. Our research indicates that the level of achievement is indeed associated with a higher probability of being involuntarily single, and it sheds light on how it is so. Most likely, it is not the case that a low level of achievement repulses prospective mates. A more likely scenario is that a low level of achievement negatively affects one’s self-esteem, and impaired self-esteem impairs flirting capacity, which in turn makes it more difficult for people to attract mates.

Consistent with the sociometer understanding of self-esteem^23,24^, we found that a higher perceived level of achievement was associated with higher self-esteem. Yet, it is likely that the effect also goes in the opposite direction: People who have high self-esteem see themselves as having achieved more in life than people who have low self-esteem. We believe that the relationship between perceived level of achievement and self-esteem is indeed bidirectional: Achievements in life boost self-esteem, but higher self-esteem also makes people see themselves as having achieved more in life. It could be argued that achievement has little impact on self-esteem and the direction of the relationship only goes from self-esteem to perceived achievement level. In this interpretation, the perceived level of achievement is simply an expression of self-esteem; however, the correlational strength between the two variables was moderate, meaning that a considerable part of the variation in perceived achievement level is not explained by variation in self-esteem. Future studies using a longitudinal design could attempt to disentangle the two effects.

One limitation of the current research is that it was based on self-report instruments that are subject to biases such as people giving inaccurate answers. Moreover, we employed a non-probability sample, so our findings may not readily generalize to the population. Similarly, our research was confined to the Greek cultural context, so our findings may not readily generalize to other cultural settings. Accordingly, future cross-cultural research is required to examine whether the observed associations hold in different cultural settings. Additionally, recent advancement in artificial intelligence has produced tools that enable us to construct efficiently sound psychometric instruments^26^. Accordingly, for the purposes of the current research, we employed AI to develop the instruments measuring desire for independence and perceived level of achievement. Yet, although our instruments appeared to have face validity and behaved in accordance with our hypotheses, because this methodology is novel, future studies need to attempt to replicate our findings using a different instrument to measure these traits.

Furthermore, as discussed previously, our study is correlational and causality between the measured variables can be assumed but not proven by our data. For instance, in the present study, we were not able to directly test mediation as our data are cross-sectional. Future longitudinal studies are needed to examine causal relationships between the identified variables. In addition, the current study found evidence that desire for independence and perceived level of achievement were associated with singlehood. Yet, these factors are likely to be predicted by other factors that we have not measured. For instance, some people may have been exposed to intimate partners who were very clingy, and this experience may have made them more appreciative of being independent. Future studies need to identify the factors that predict desire for independence and perceived level of achievement and investigate how they relate to singlehood. Moreover, in the current study, there was no option for participants to report transgender or non-binary status, and sexual orientation was not recorded. Future replication studies, need to address these issues.

Our results indicate that people who have a high desire for independence are also more likely to be voluntarily single. In addition, people who perceive themselves to have achieved few things in life are likely to have low self-esteem, decreased capacity for flirting, and an increased probability of being involuntarily single. Future studies are needed, however, to better understand the relationship of these variables with singlehood.

## Appendix

### Desire for independence

The desire for independence instrument consists of nine items listed below. The mean total score is calculated by finding the average score.

Participants were asked to “Rate how you see yourself by rating the following statements:” and they were subsequently provided with the nine items to rate in the following scale: 1 – Strongly disagree, 5 – Strongly agree.

1. I prefer to be able to make my own decisions in life.
2. I like being free to do whatever I want.
3. I like to feel in control of my life.
4. I wouldn’t trade my independence for anything.
5. I like having the freedom to plan my own activities and schedule.
6. I don’t like others controlling my movements.
7. I prefer to live life on my own terms, even if it means facing difficulties.
8. I highly value my personal freedom and independence.
9. I don’t feel good if I don’t have my own space.

#### Perceived level of achievement

The perceive level of achievement consist of three items listed below. The mean total score is calculated by reverse-scoring the third item and finding the average score.

Participants were asked to “Rate how you see yourself by rating the following statements:” and they were subsequently provided with the three items to rate in the following scale: 1 – Strongly disagree, 5 – Strongly agree.

1. I have achieved many things in my life.
2. Given where I started, I think I’ve come pretty far.
3. I haven’t achieved enough things in my life to be proud of.

#### Zero-order correlation matrix

The correlation matrix below was produced using Pearson’s correlation statistic.

**Table:** 

	Desire for independence	Perceived level of achievement	Flirting capacity	Self-esteem
Desire for independence	1	0.167^**^	0.129^**^	0.161^**^
		< 0.001	< 0.001	< 0.001
Perceived level of achievement	0.167^**^	1	0.335^**^	0.643^**^
	< 0.001		< 0.001	< 0.001
Flirting capacity	0.129^**^	0.335^**^	1	0.491^**^
	< 0.001	< 0.001		< 0.001
Self-esteem	0.161^**^	0.643^**^	0.491^**^	1
	< 0.001	< 0.001	< 0.001	

** Correlation is significant at the 0.01 level (2-tailed).

## Data Availability

All data are available here: https://osf.io/jkzyb/?view_only=f8d0d6746bfc4f36a5903886f8e627f3.
